# Status of Electromagnetically Accelerating Universe

**DOI:** 10.3390/e26080629

**Published:** 2024-07-26

**Authors:** Paul H. Frampton

**Affiliations:** Dipartimento di Matematica e Fisica “Ennio De Giorgi”, Università del Salento and INFN-Lecce, Via Arnesano, 73100 Lecce, Italy; paul.h.frampton@gmail.com

**Keywords:** accelerated expansion, dark matter, electromagnetism

## Abstract

To describe the dark side of the universe, we adopt a novel approach where dark energy is explained as an electrically charged majority of dark matter. Dark energy, as such, does not exist. The Friedmann equation at the present time coincides with that in a conventional approach, although the cosmological “constant” in the Electromagnetic Accelerating Universe (EAU) Model shares a time dependence with the matter component. Its equation of state is *ω* ≡ *P*/*ρ* ≡ −1 within observational accuracy.

## 1. Introduction to the EAU Model

Theoretical cosmology is at an exciting stage because about 95% of the energy in the Visible Universe remains incompletely understood. The 25% which is dark matter has constituents whose mass is unknown by over one hundred orders of magnitude. The 70% which is dark energy is, if anything, more mysterious. Although it can be parametrised by a cosmological constant with an equation of state ω=−1, which provides an excellent phenomenological description, that is only a parametrisation and not a complete understanding.

In the present paper, we address the issues of dark matter and dark energy using a novel approach. We use only the classical theories of electrodynamics and general relativity. We shall not employ any knowledge of quantum mechanics or of theories describing short-range strong and weak interactions.

This paper may be regarded as a follow-up to our 2018 paper [1] entitled *On the Origin and Nature of Dark Matter* and we could have simply added *and Energy* to that title. We have, however, chosen *Status of Electromagnetic Accelerating Universe* because it more accurately characterises our present emphasis on the EAU model whose main idea is that electromagnetism dominates over gravitation in the explanation of the accelerating cosmological expansion. This idea takes us beyond the first paper [2] that applied general relativity to theoretical cosmology. This is not surprising, since in 1917, that author was obviously unaware of the fact [3,4] that is was discovered only in 1998 that the rate of cosmological expansion is accelerating.

The make up of this paper is that primordial black holes are discussed in Section 2, then primordial naked singularities are discussed in Section 3. Finally, in Section 4, there is a discussion.

## 2. Primordial Black Holes (PBHs)

Black holes may be classified into those which arise from the gravitational collapse of stars and others, which do not. We shall refer to all of the others as primordial. In general, PBHs with masses up to $10^{5}M_{⊙}$ are expected to be formed during the first second after the Big Bang and arise from inhomogeneities and fluctuations of spacetime. The existence of PBHs was first proposed [5] by Novikov and Zeldovich and independently seven years later in the West by Carr and Hawking [6]. The idea that dark matter constituents are PBHs was first suggested by Chapline [7].

Shortly after the original presentation of general relativity [8,9,10], a metric describing a static black hole of mass M with zero charge and zero spin was discovered by Schwarzschild [11] in the form

(1) $$ds^{2}=-\left(1-\frac{r_{S}}{r}\right)dt^{2}+{\left(1-\frac{r_{S}}{r}\right)}^{-1}dr^{2}+r^{2}d\Omega^{2}$$

Shortly thereafter, the Reissner–Nordstrom metric [12,13] for a static black hole with electric charge was found. It then took a surprising forty-five years until Kerr cleverly found a metric [14] of general relativity corresponding to such a solution with spin. We shall not discuss the case of non-zero spin in the present paper because, although we expect that all the objects we discuss do spin in nature, according to the calculations in [15], which use Kerr’s generalisation, spin is an inessential complication in all of our subsequent considerations.

### 2.1. Primordial Intermediate Mass Black Holes (PIMBHs) as Galactic Dark Matter

Global fits to cosmological parameters have led to a consensus that about one quarter of the energy of the universe is in the form of electrically neutral dark matter. It seemed natural to propose [16] that in a galaxy like the Milky Way are between ten million and ten billion primordial black holes with masses between one hundred and one hundred thousand solar masses.

Black holes in this range of masses are naturally known as intermediate mass black holes (IMBHs) since they lie as an intermediate between the masses of stellar mass black holes and the masses of the supermassive black holes at galactic centers.

The existence of stellar mass black holes in nature was established sixty years ago in 1964 by the discovery in Cygnus X-1 of a black hole with a mass of about $15M_{⊙}$. Such X-ray binaries were studied in [17] and then in [18] and appear in the mass range between $5M_{⊙}$ and $100M_{⊙}$.

The existence of dark matter was first discovered by Zwicky [19,20] in 1933 in the Coma Clusters and its presence in individual galaxies was demonstrated convincingly by Rubin in the 1970s from the measurement of rotation curves, which demanded the existence of additional matter to what was luminous [21].

The PBH mass function is all important. Possible PBH masses extend upwards to many solar masses and without any obvious upper limit, far beyond what was thought possible in the twentieth century, when ignorance about PBHs with many solar masses probably prevented the MACHO [22] and EROS [23] collaborations from discovering a larger fraction of dark matter.

Black holes formed by gravitational collapse cannot satisfy $M_{BH}\ll M_{⊙}$ because stars powered by nuclear fusion cannot be far below $M=M_{⊙}$. This was contradicted by the studies in [5,6], which suggested that much lighter black holes can be produced in the earliest stages of the Big Bang.

Such PBHs are of special interest for several reasons. Firstly, they are the only type of black hole that can be so light, down to $10^{12}$ kg $∼10^{-18}M_{⊙}$, that Hawking radiation might conceivably be detected. Secondly, PBHs in the intermediate mass region $100M_{⊙}\leq M_{IMBH}\leq 10^{5}M_{⊙}$ can provide galactic dark matter.

The mechanism of PBH formation involves large fluctuations or inhomogeneities. Carr and Hawking [6] argued that we know there are fluctuations in the universe in order to seed structure formation and there must similarly be fluctuations in the early universe. Provided the radiation is compressed to a high density, meaning to a radius as small as its Schwarzschild radius, a PBH will form. Because the density in the early universe is extremely high, it is very likely that PBHs will be created. The two necessities are high density, which is guaranteed, and large inhomogeneities, which are possible.

During radiation domination,

(2) $$a\left(t\right)\propto t^{1/2}$$

and

(3) $$\rho_{\gamma }\propto a{\left(t\right)}^{-4}\propto t^{-2}$$

Ignoring factors O(1) and bearing in mind that the radius of a black hole is

(4) $$r_{BH}∼\left(\frac{M_{BH}}{M_{Planck}^{2}}\right)$$

with

(5) $$M_{Planck}∼10^{19}GeV∼10^{-8}kg∼10^{-38}M_{⊙}$$

and using the Planck density $\rho_{Planck}$

(6) $$\rho_{Planck}\equiv {\left(M_{Planck}\right)}^{4}∼\left(10^{-5}g\right){\left(10^{-33}cm\right)}^{-3}=10^{94}\rho_{H_{2}O}$$

the density of a general black hole $\rho_{BH}\left(M_{BH}\right)$ is

(7) $$\rho_{BH}\left(M_{BH}\right)∼\left(\frac{M_{BH}}{r_{BH}^{3}}\right)=\rho_{Planck}{\left(\frac{M_{Planck}}{M_{BH}}\right)}^{2}∼10^{94}\rho_{H_{2}O}{\left(\frac{10^{-38}M_{⊙}}{M_{BH}}\right)}^{2}$$

which means that for a solar mass black hole

(8) $$\rho_{BH}\left(M_{⊙}\right)∼10^{18}\rho_{H_{2}O}$$

while for a billion solar mass black hole

(9) $$\rho_{BH}\left(10^{9}M_{⊙}\right)∼\rho_{H_{2}O}.$$

and above this mass, the density falls as $M_{BH}^{-2}$.

The mass of the PBH is derived by combining Equations (3) and (7). We see from these two equations that $M_{PBH}$ grows linearly with time, and using Planckian units or solar units, we find, respectively,

(10) $$M_{PBH}∼\left(\frac{t}{10^{-43}sec}\right)M_{Planck}∼\left(\frac{t}{1sec}\right)10^{5}M_{⊙}$$

which implies that if we insisted on PBH formation before the electroweak phase transition, $t<10^{-12}s$, that

(11) $$M_{PBH}<10^{-7}M_{⊙}$$

Such an upper bound as that in Equation (11) explains why the MACHO searches at the turn of the twenty-first century [22,23], inspired by the clever suggestion of Paczynski [24], lacked motivation to pursue searching above $100M_{⊙}$, because it was thought incorrectly at that time that PBHs were far too light. It was known correctly that the results of the gravitational collapse of normal stars, or even large early stars, are below $100M_{⊙}$. Supermassive black holes with $M>10^{6}M_{⊙}$ such as $SgrA^{*}$ in the Milky Way were beginning to be discovered in galactic centers but their origin was unclear and this will be discussed further in Section 2.2.

Using the mechanism for Hawking radiation provides the lifetime for a black hole evaporating *in vacuo* given by

(12) $$\tau_{BH}∼{\left(\frac{M_{BH}}{M_{⊙}}\right)}^{3}\times 10^{64}years$$

so that to survive to the age $10^{10}$ years of the universe, there is a lower bound on $M_{PBH}$ to augment the upper bound in Equation (11), giving, as the full range of Carr–Hawking PBHs,

(13) $$10^{-18}M_{⊙}<M_{PBH}<10^{-7}M_{⊙}$$

The lowest mass possible for s surviving PBH in Equation (13) has the density $\rho ∼10^{58}\rho_{H_{2}O}$. It is an object which has the physical size of a proton and the mass of Mount Everest.

The Hawking temperature $T_{H}\left(M_{BH}\right)$ of a black hole is given by

(14) $$T_{H}\left(M_{BH}\right)=6\times 10^{-8}K\left(\frac{M_{⊙}}{M_{BH}}\right)$$

which would be above the CMB temperature, and hence there would be outgoing radiation for all of the cases with $M_{BH}<2\times 10^{-8}M_{⊙}$. Hypothetically, if the dark matter halo were made entirely of the brightest possible (in terms of Hawking radiation) $10^{-18}M_{⊙}$ PBHs, the expected distance to the nearest PBH would be about $10^{7}$ km. Although the PBH temperature, according to Equation (14), is $∼6\times 10^{10}K$, the inverse square law renders the intensity of Hawking radiation too small, by many orders of magnitude, to allow for detection by any foreseeable terrestrial apparatus.

The originally suggested mechanism produces PBHs with masses in the range up to $10^{-7}M_{⊙}$. We shall now discuss the formation of far more massive PBHs by a rather different mechanism. As already discussed, PBH formation requires very large inhomogeneities. Here, we shall illustrate how mathematically to produce inhomogeneities that are exponentially large.

In the simplest single-stage inflation, no exceptionally large-density perturbation is expected. Therefore, it is necessary to consider at least a two-stage hybrid inflation with respective fields called [25], inflaton, and waterfall. The idea then involves parametric resonance in that, after the first of the two stages of inflation, mutual couplings of the inflaton and waterfall fields cause both to oscillate arbitrarily wildly and produce perturbations which can grow exponentially. A second (waterfall) inflation then stretches the inhomogeneities further, thus enabling the production of PBHs with an arbitrarily high mass. This specific model may not describe nature but provides an existence theorem to confirm that arbitrarily large-mass PBHs can be produced mathematically. The resulting mass function is spiked, but it is possible that other PBH production mechanisms can produce a smoother mass function.

The full details of the model are presented in [26], where the inflaton and waterfall fields are denoted by σ and ψ, respectively. Between the two stages of inflation, the σ and ψ fields oscillate, decaying into their quanta via their own and mutual couplings. Specific modes of σ and ψ are amplified by parametric resonance. The resulting coupled equations for the two fields are of the Mathieu type with exponentially growing solutions. The numerical solution shows that the peak wave number $k_{peak}$ is approximately linear in $m_{\sigma }$. The resultant PBH mass, the horizon mass when the fluctuations re-enter the horizon, is approximately

(15) $$M_{PBH}∼1.4\times 10^{13}M_{⊙}{\left(\frac{k_{peak}}{Mpc^{-1}}\right)}^{-2}$$

Explicit plots were exhibited in [26] for the cases $M_{PBH}=10^{-8}M_{⊙},10^{-7}M_{⊙}$ and $10^{5}M_{⊙}$. At that time (2010), although not included in the paper, it was confirmed that parameters can always be chosen such that arbitrarily high-mass PBHs, at or even beyond the mass of the universe, may be produced. This is an important result to be borne in mind.

In the PBH production mechanism based on hybrid inflation with parametric resonance, the mass function is generally sharply spiked at a specific mass region. Such a peculiar mass function is not expected to be a general feature of PBH formation, only a property of this specific mechanism. But this specific mechanism readily demonstrates the possibility of the primordial formation of black holes with many solar masses. For completeness, it should be pointed out that PBHs with masses up to $10^{-15}M_{⊙}$ were discussed even in the 1970s, for example, by Carr [27] and by Novikov, Polnarev, Starobinskii, and Zeldovich [28].

For dark matter in galaxies, PIMBHs are important, where the upper end must be truncated at $10^{5}M_{⊙}$ to stay well away from galactic disk instability, first discussed by Ostriker et al [29]. They showed convincingly that an object with a mass one million solar masses out in the spiral arms of the Milky Way destabilizes the galactic disk to such an extent that the entire galaxy collapses.

The observations of rotation curves reveal that the dark matter in galaxies including the Milky Way fills out an approximately spherical halo somewhat larger in radius than the disk occupied by the luminous stars. Numerical simulations of structure formation suggest a profile of the dark matter of the NFW type [30]. Note that the NFW profile is independent of the mass of the dark matter constituent and the numerical calculations are restricted by the available computer size, for a system as large as a typical galaxy, to constituents which have many solar masses.

In our discussion a decade ago [16], we focused on galaxies like the Milky Way and restricted the mass range for dark matter constituents to lie within three orders of magnitude:

(16) $$10^{2}M_{⊙}<M<10^{5}M_{⊙}$$

We shall not repeat the lengthy entropy arguments in [16] here, just that the constituents were proposed to be primordial intermediate mass black holes, PIMBHs.

Assuming a total dark halo mass of $10^{12}M_{⊙}$, Equation (16) implies that the number of PIMBHs is between ten million ($10^{7}$) and ten billion ($10^{10}$). Assuming further that the dark halo has a radius *R* of a hundred thousand ($10^{5}$) light years, the mean separation $\bar{L}$ of PIMBHs can then estimated by

(17) $$\bar{L}∼\left(\frac{R}{N^{1/3}}\right)$$

which translates approximately to

(18) $$100ly<\bar{L}<1000ly$$

which also provides a reasonable estimate of the distance to the nearest PIMBH from the Earth, which is very far outside the Solar System where the orbital radius of the outermost planet Neptune is ∼0.001 ly.

To an outsider, it may be surprising that millions of intermediate mass black holes in the Milky Way have remained undetected. Ironically, they could have been detected more than two decades ago had the MACHO collaboration [22] persisted in its microlensing experiment at Mount Stromlo Observatory in Australia.

Dark matter was first discovered almost a century ago by Zwicky [19,20] in the Coma cluster, a large cluster at 99 Mpc containing over a thousand galaxies and with a total mass estimated at $6\times 10^{14}M_{⊙}$ [31]. Convincing proof of the existence of cluster dark matter was provided by the Bullet cluster collision, where the distinct behaviours of the X-ray-emitting gas which collided, and the dark matter which did not, was observable [32,33,34].

Since there is not the same disk stability limit [29] as for galaxies, the constituents of cluster dark matter can also involve PSMBHs up to much higher masses than those possible for the PIMBHs within galaxies.

The possible solution of the galactic dark matter problem cries out for experimental verification. Three methods have been discussed: wide binaries, distortion of the CMB, and microlensing. Of these, microlensing seems the most direct and promising. Microlensing experiments were carried out by the MACHO [22] and EROS [23] collaborations decades ago. At that time, it was believed that PBH masses were below $10^{-7}M_{⊙}$ by virtue of the Carr–Hawking mechanism. Heavier black holes could, it was then believed, arise only from the gravitational collapse of normal stars, or heavier early stars, and would have a mass below $100M_{⊙}$.

For this reason, there was no motivation to suspect that there might be MACHOs which led to higher-duration microlensing events. The longevity, $\hat{t}$, of an event is

(19) $$\hat{t}=0.2yrs{\left(\frac{M_{PBH}}{M_{⊙}}\right)}^{\frac{1}{2}}$$

which assumes a transit velocity of 200 km/s. Subsituting our extended PBH masses, one finds approximately $\hat{t}∼6,20,60$ years for $M_{PBH}∼10^{3},10^{4},10^{5}M_{⊙}$, respectively, and searching for light curves with these higher values of $\hat{t}$ could be rewarding.

It is to be hoped that MACHO searches will soon resume at the Vera Rubin Observatory and focus on highest-longevity microlensing events. Is it possible that convincing observations showing only a fraction of a light curve could suffice? If so, only a fraction of the six years, for example, corresponding to PIMBHs with one thousand solar masses, could be enough to confirm the theory.

### 2.2. Primordial Supermassive Black Holes (PSMBHs) at Galactic Centers

Evidence for supermassive black holes at galactic centers arises from the observations of fast-moving stars around them and such stars being swallowed or torn apart by the strong gravitational field. The first discovered SMBH was Sgr $A^{*}$, at the core of the Milky Way, which was discovered in 1974 and has a mass $M_{SgrA*}∼4.1\times 10^{6}M_{⊙}$. The SMBH at the core of the nearby Andromeda galaxy (M31) has a mass $M=2\times 10^{8}M_{⊙}$, fifty times $M_{SgrA*}$. The most massive core SMBH so far observed is for NGC4889, with a mass of $M∼2.1\times 10^{9}M_{⊙}$. Some galaxies contain two SMBHs in a binary, expected to be the result of a galaxy merger. Quasars contain black holes with even higher masses up to at least $4\times 10^{10}M_{⊙}$.

A black hole with the mass of that of $SgrA^{*}$ would disrupt the disk dynamics [29] were it out in the spiral arms, but at, or near to, the center of mass of the Milky Way, it is more stable. $SgrA^{*}$ is far too massive to have been the result of a gravitational collapse, and if we take the view that all black holes either are the result of gravitational collapse or are primordial, then the galaxies’ core SMBHs must be primordial. Nevertheless, it is probable that the PSMBHs are built up by merging and accretion from less massive PIMBH seeds.

## 3. Primordial Naked Singularities (PNSs)

Just as neutral black holes can be formed as PBHs in the early universe, it is natural to assume that objects can be formed based on the Reissner–Nordstrom metric [12,13]:

(20) $$ds^{2}=f\left(r\right)dt^{2}-f{\left(r\right)}^{-1}dr^{2}-r^{2}d\theta^{2}-r^{2}\sin^{2}\theta d\phi^{2}$$

where

(21) $$f\left(r\right)\equiv \left(1-\frac{r_{S}}{r}+\frac{r_{Q}^{2}}{r^{2}}\right).$$

with

(22) $$r_{S}=2GM\;\;\;\;\;\;r_{Q}=Q^{2}G$$

The horizon(s) of the RN metric occur when

(23) f(r)=0

which gives

(24) $$r_{\pm }=\frac{1}{2}\left(r_{S}\pm \sqrt{r_{S}^{2}-4r_{Q}^{2}}\right)$$

It follows that for $2r_{Q}<r_{S}$, $Q^{2}<M$, there are two horizons. On the other hand, when $2r_{Q}=r_{S}$, $Q^{2}=M$, the RN black hole is named extremal and there is only one horizon. If $2r_{Q}>r_{S}$, $Q^{2}>M$, the RN metric may be called super-extremal. In this case, there is no horizon at all and the r=0 singularity becomes observable to a distant observer. This is called a naked singularity. With this last inequality, it is no longer a black hole, which, by definition, requires a horizon.

Consider two identical objects with mass M and charge Q, and then an electromagnetic repulsive force $F_{em}\propto k_{e}Q^{2}$ and a gravitational attraction $F_{grav}\propto GM^{2}$. Thus, for the electromagnetic repulsion to exceed the gravitational attraction, we need $Q^{2}>GM^{2}/k_{e}$ and hence perhaps super-extremal Reissner–Nordstrom or naked singularities (NSs) (to anticipate NSs, we shall replace BH with NS for charged dark matter. If charges satisfy $Q^{2}<M$, this replacement is unnecessary).

We cannot claim to understand the formation of PNSs. One idea hinted at in [35] is that extremely massive ones, charged PEMNSs, might begin life as electrically neutral PBHs. Then, during the dark ages, these selectively accrete electrons over protons. However this formation process evolves, it must be completed before the onset of accelerated expansion some 4 billlion years ago at cosmic time t∼9.8 Gy.

### Like-Sign-Charged Primordial Extremely Massive Naked Singularities (PEMNSs) and Accelerated Expansion: The EAU Model

A novel EAU model was suggested in [36,37], where dark energy is replaced by charged dark matter in the form of PEMNSs or charged primordial extremely massive naked singularities (in [36,37] the PEMNSs were called PEMBHs). That discussion involved the new idea that, at the very largest cosmological distances, the dominant force is electromagnetism rather than gravitation. This differs from the assumption tacitly made in the first application of general relativity by Einstein [2].

The production mechanism for PBHs in general is not well understood, and for the PEMNSs, we shall make the assumption that they are formed before the accelerated expansion begins at $t=t_{DE}∼9.8$ Gy. For the expansion before $t_{DE}$, we shall assume that the ΛCDM model is approximately accurate.

The subsequent expansion in the charged dark matter model will, in the future, depart markedly from the ΛCDM case. We can regard this as advantageous because the future fate of the universe in the conventional picture does have certain unaesthetic features in terms of the extremely large size of the asymptotic extroverse.

In the ΛCDM model, the introverse, or what is also called the visible universe, coincides with the extroverse at $t=t_{DE}∼9.8$ Gy with the common radius

(25) $$R_{EV}\left(t_{DE}\right)=R_{IV}\left(t_{DE}\right)=39Gly.$$

The introverse expansion is limited by the speed of light and its radius increases from Equation (25) to 44 Gly at the present time $t=t_{0}$, but asymptotes only to

(26) $$R_{IV}\left(t\to \infty \right)\to 58Gly$$

The extroverse expansion is, by contrast, exponential and superluminal. Its radius increases from its value of 39 Gly in Equation (25) to 52 Gly at the present time $t=t_{0}$ and grows without limit. After only a trillion years, it attains an extremely large value, as follows:

(27) $$R_{EV}\left(t=1Ty\right)=9.7\times 10^{32}Gly.$$

This future for the ΛCDM scenario seems distasteful because the introverse becomes of ever decreasing, and eventually vanishing, significance, relative to the extroverse.

One attempt at a possible formation mechanism of PEMNSs was provided in [35], where their common sign of electric charge, negative, arises from the preferential accretion of electrons relative to protons. This formation mechanism is not well understood (electrically neutral PEMBHS were first considered, with a different acronym, SLABs, in [38]). So, to create a cosmological model, we shall, for simplicity, assume that the PEMNSs are all formed before $t=t_{DE}∼9.8$ Gy and thereafter, the Friedmann equation, ignoring radiation, is

(28) $${\left(\frac{\dot{a}}{a}\right)}^{2}=\frac{\Lambda (t)}{3}+\frac{8\pi G}{3}\rho_{matter}$$

where Λ(t) is the cosmological “constant” generated by Coulomb repulsion between the PEMNSs. From Equation (28), in the ΛCDM model with $a(t_{0})=1$ and constant $\Lambda \left(t\right)\equiv \Lambda_{0}$, we would predict that, in the distant future,

(29) $$a\left(t\to \infty \right)∼exp\left(\sqrt{\frac{\Lambda_{0}}{3}}\left(t-t_{0}\right)\right)$$

In the case of charged dark matter, with no dark energy, we must re-write Equation (28) as

(30) $${\left(\frac{\dot{a}}{a}\right)}^{2}=\frac{8\pi G}{3}\rho_{cPEMNSs}+\frac{8\pi G}{3}\rho_{matter}$$

in which

(31) $$\rho_{matter}\left(t\right)=\frac{\rho_{matter}\left(t_{0}\right)}{a{\left(t\right)}^{3}}$$

where matter includes normal matter and uncharged dark matter.

Of special interest to the present discussion is the expected future behaviour of the charged dark matter:

(32) $$\rho_{PEMNSs}\left(t\right)=\frac{\rho_{PEMNSs}\left(t_{0}\right)}{a{\left(t\right)}^{3}}$$

so that the comparison of Equations (28) and (30) suggests that the cosmological constant is predicted to decrease from its present value. More specifically, we find that, asymptotically, the scale factor will behave as if matter-dominated and the cosmological constant will decrease at large future times as a power:

(33) $$a\left(t\to \infty \right)∼t^{\frac{2}{3}}\;\;\;\;\;\;\Lambda \left(t\to \infty \right)∼t^{-2}.$$

so that a trillion years in the future, Λ(t) will have decreased by some four orders of magnitude relative to $\Lambda (t_{0})$. See Table 1.

In both the ΛCDM model and the EAU model, the present time is an unusual one in cosmic history. In the former case, there is the present similarity between the the densities of dark matter and energy. In the latter case with charged dark matter, the present accelerated expansion is maximal and will disappear within a few more billion years.

In the EAU model, acceleration began about 4 Gy ago at $t_{DE}=9.8Gy=t_{0}-4Gy$. This behaviour will disappear in a few more billion years. The value of the cosmological constant is predicted to fall like $a{\left(t\right)}^{-2}$ so that, when $t∼\sqrt{2}t_{0}∼19.5Gy∼t_{0}+4.7Gy$, the value of Λ(t) will be one half of its present value, $\Lambda (t_{0})$. On the other hand, as discussed in [37], the equation of state associated with Λ is accurately predicted to be ω=−1, so close to that value that measuring the difference seems forever impracticable.

For charged dark matter, we now discuss the future time evolution of the introverse and extroverse. For the introverse, nothing changes from the ΛCDM, and after a trillion years, the introverse radius will be at its asymptotic value $R_{IV}=58Gly$, as stated in Equation (26). By contrast, the future for the extroverse is very different for charged dark matter than for the conventional ΛCDM case. With the growth $a\left(t\right)\propto t^{\frac{2}{3}}$, we find that the radius of the extroverse at t=1 Ty is

(34) $$R_{EV}\left(t=1Ty\right)∼900Gly.$$

This is in stark contrast to the extremely large value $9.7\times 10^{32}$ Gly predicted by the ΛCDM model, quoted in Equation (27) above. Equation (34) means that if there still exist scientific observers, their view of the distant universe will be quite similar to that of the present one and will include many billions of galaxies.

In the ΛCDM case, such a hypothetical observational cosmologist, trillions of years in the future, could observe only the Milky Way and objects which are gravitationally bound to it, so that cosmology would become an extinct science.

The principal physics advantage of charged dark matter is that it avoids the idea of an unknown repulsive gravity inherent in “dark energy”. Electromagnetism provides the only known long-range repulsion so it is more attractive to adopt it as the explanation for the accelerating universe. The secondary advantage of charged dark matter, that it provides a conducive environment for observational cosmology trillions of years into the future, is not by itself sufficient to choose this theory.

## 4. Discussion

Although this paper is essentially speculative, we are unaware of any fatal flaw. We have replaced the conventional make up for the slices of the universe’s energy pie (5% normal matter; 25% dark matter; 70% dark energy) with a similar but crucially changed version (5% normal matter; 25% dark matter; 70% charged dark matter).

The term dark energy was coined by Turner [39] in 1998, shortly after the announcement of accelerated expansion [3,4]. An outsider familiar with $E=Mc^{2}$ might guess that dark energy and matter are equivalent. If our model is correct, they would be correct, although it has nothing to do with $E=mc^{2}$. Charged dark matter replaces dark energy, an ill-chosen name because it suggested that there exists an additional component in the Universe.

In April 2024, news [40] from the Dark Energy Spectroscopic Instrument (DESI) at Kitt Peak in Arizona, USA, gave a preliminary indication that the cosmological constant Λ(t) is not constant but diminishing with time, as suggested by our Equation (33), and by our Table 1, thus providing possible support for the EAU model.

Other supporting evidence could appear in the foreseeable future from the James Webb Space Telescope (JWST), which might shed light on the formation of PBHs in the early universe, and also from the Vera C. Rubin Observatory in Chile, which will study long-duration microlensing light curves, which could provide evidence for the existence of PIMBHs inside the Milky Way.

It will be interesting to learn how these and other observations might support the idea that the observed cosmic acceleration is caused by charged dark matter.

## Figures and Tables

**Table 1:** Cosmological “constant”.

Time	Λ(t)
$t_{0}$	(2.0 ${meV)}^{4}$
$t_{0}+10Gy$	(1.0 ${meV)}^{4}$
$t_{0}+100Gy$	(700 ${\mu eV)}^{4}$
$t_{0}+1Ty$	(230 ${\mu eV)}^{4}$
$t_{0}+1Py$	(7.4 ${\mu eV)}^{4}$

## Data Availability

No new data were created or analyzed in this study. Data sharing is not applicable to this article.
